# Implementation and Clinical Utility of Suicide Crisis Syndrome Screening in an Acute Psychiatric Setting: A Prospective Observational Study

**DOI:** 10.1192/j.eurpsy.2026.12072

**Published:** 2026-07-31

**Authors:** C. Lovig, P. Osváth, C. Molnár, F. N. Major, S. Venczák, S. Fekete, T. Tényi, L. Cohen, I. Galynker, V. Vörös

**Affiliations:** 1 Department of Psychiatry and Psychotherapy; 2 Medical School, University of Pécs, Pécs, Hungary; 3 Mount Sinai Suicide Prevention Research Laboratory, Icahn School of Medicine at Mount Sinai, New York, United States

## Abstract

**Introduction:**

Suicide is still a major public health challenge with approximately 800,000 deaths annually worldwide. Traditional risk assessments heavily rely on self-reported suicidal ideation and static risk factors, which may be insufficient for detecting imminent suicide risk. Suicide Crisis Syndrome (SCS), defined as a pre-suicidal mental-state characterized by affective and cognitive dysregulation may be novel helpful tool in complex suicide risk assessment.

**Objectives:**

This study aimed to implement SCS screening protocols in an acute psychiatric setting and to evaluate their impact on clinical decision-making and patient pathways.

**Methods:**

A prospective observational study was conducted in a university hospital between January and June, 2024. All patients admitted to the acute inpatient and outpatient psychiatric units were assessed using a three-tier screening model. Patients screened positive for SCS underwent structured diagnostic evaluation using the SCS-C (Suicide Crisis Syndrome Checklist) checklist. The associations between SCS symptoms, clinical decisions (admission vs. outpatient care) and treatment outcomes were analysed.

**Results:**

Of 668 patients screened, SCS positivity showed strong predictive value for inpatient admission. 70% of admitted patients screened positive for SCS, compared to only 30% among outpatients. Core SCS symptoms (entrapment, cognitive dysregulation, and social withdrawal) were most predictive for hospitalization. The A-SCS-C demonstrated excellent psychometric performance (sensitivity: 0.955, specificity: 0.723) in identifying cases confirmed by the SCS-C. A significant decrease in SCS symptom burden was observed during hospitalization, particularly between days 10 and 20, indicating treatment response.

**Conclusions:**

SCS screening provide a valuable framework for detecting acute suicidal states that may be overlooked by conventional assessments. A-SCS-C has high sensitivity and specificity with SCS diagnostic tool. The SCS-C may function as a clinical decision-making support tool, however further longitudinal research is warranted to evaluate long-term outcomes, including readmission and suicide prediction.

**Disclosure of Interest:**

None Declared

